# Enhancing EDM Machining Precision through Deep Cryogenically Treated Electrodes and ANN Modelling Approach

**DOI:** 10.3390/mi14081536

**Published:** 2023-07-31

**Authors:** Kashif Ishfaq, Muhammad Sana, Muhammad Umair Waseem, Waqar Muhammad Ashraf, Saqib Anwar, Jaroslaw Krzywanski

**Affiliations:** 1 Department of Industrial and Manufacturing Engineering, University of Engineering and Technology, Lahore 54890, Pakistanumair.uw1@gmail.com (M.U.W.); 2 Sargent Centre for Process Systems Engineering, Department of Chemical Engineering, University College London, Torrington Place, London WC1E 7JE, UK; 3 Industrial Engineering Department, College of Engineering, King Saud University, P.O. Box 800, Riyadh 11421, Saudi Arabia; sanwar@ksu.edu.sa; 4 Department of Advanced Computational Methods, Jan Dlugosz University in Czestochowa, 42-200 Czestochowa, Poland

**Keywords:** Inconel 617, EDM, cryogenically, overcut, Cu, brass

## Abstract

The critical applications of difficult-to-machine Inconel 617 (IN617) compel the process to be accurate enough that the requirement of tight tolerances can be met. Electric discharge machining (EDM) is commonly engaged in its machining. However, the intrinsic issue of over/undercut in EDM complicates the achievement of accurately machined profiles. Therefore, the proficiency of deep cryogenically treated (DCT) copper (Cu) and brass electrodes under modified dielectrics has been thoroughly investigated to address the issue. A complete factorial design was implemented to machine a 300 μm deep impression on IN617. The machining ability of DCT electrodes averagely gave better dimensional accuracy as compared to non-DCT electrodes by 13.5% in various modified dielectric mediums. The performance of DCT brass is 29.7% better overall compared to the average value of overcut (OC) given by DCT electrodes. Among the non-treated (NT) electrodes, the performance of Cu stands out when employing a Kerosene-Span-20 modified dielectric. In comparison to Kerosene-Tween-80, the value of OC is 33.3% less if Kerosene-Span-20 is used as a dielectric against the aforementioned NT electrode. Finally, OC’s nonlinear and complex phenomena are effectively modeled by an artificial neural network (ANN) with good prediction accuracy, thereby eliminating the need for experiments.

## 1. Introduction

Machining with precise and effective geometric or dimensional accuracy has remained of great interest to researchers for the last few decades. The design and production of molds and dies within the manufacturing sector with greater dimensional accuracy has been challenging for manufacturers and researchers [1]. Due to their strength, Nickel (Ni)-based superalloys, titanium alloys, and other alloys are hard to cut with conventional machining. Thereof, non-conventional machining processes are recommended as the potential choice for the cutting of hard-to-machine superalloys [2]. Poor geometric precision is still a challenge, even though non-traditional machining procedures are preferred [3]. Difficult to cut Ni-based superalloys, indubitably, IN617 has earned marvelous popularity in many fields, such as combustion cans, super boilers, aircraft, and aerospace [4]. The various properties of the said Ni superalloy, i.e., low density, greater strength, high oxidative and rust resistance, and stability at the upper extreme cutting environment, have made it an easy selection for the use of the abovementioned areas [5]. The conventional ways of cutting Ni-based superalloys, i.e., milling, drilling, and lathe, seem improper due to their unique characteristics like high strength, low thermal conductivity, and rapid strain hardening [6]. Thereof, a non-traditional way of machining has been built up for the cutting of the IN617. Due to the challenging environment, EDM is chosen over other non-traditional machining operations due to its greater adaptability. One of the prodigious benefits of EDM is that it can machine hard-to-cut superalloys with greater precision as well as excellent dimensional accuracy, which cannot be obtained with other traditional machining operations [7,8]. Regardless of their mechanical qualities, EDM is able to treat materials such as superalloys [9].

EDM, being a spark erosion process, has been widely accepted as a non-conventional process compared to the other older machining operations due to its capability to produce intricate shapes, dies, and molds with greater precision irrespective of the material type [10,11]. Researchers have found that EDM has the supremacy to machine hard-to-cut alloys, composites, and tough metals without introducing changes in their metallurgical characteristics [12]. EDM is a spark erosion process; as previously said, a succession of recurring sparks is used between the tool and the workpiece [13], which erodes the material from the workpiece by worsening the melting and vaporization. In order to create a discharge gap between the tool and the workpiece, the electrode, and the workpiece are submerged in the dielectric fluid, which is then ionized by the sparks that are continuously passing through it [14]. The complex shape that the electrode imprinted on the material of the workpiece is exactly the opposite of the geometry of the tool [15]. The dielectric fluid that is used in EDM operations is an extremely important factor [16]. The dielectric fluid performs a series of roles, i.e., it can: (i) flush away the debris from the workpiece material; (ii) cool down the material and the electrode during the pulse of time [17]. Commonly, kerosene oil is used for the EDM of various base materials because it is termed the traditional dielectric fluid [18]. But kerosene oil has a lower flash point and breakdown voltage, so poor output responses are achieved. Therefore, to overcome the poor material removal rate and geometrical inaccuracy, certain additives are added to the dielectric, which incorporate the dielectric, enhance the dimensional accuracy, lower the tool wear rate, and increase the surface finish of the machined specimen.

To reduce the dielectric fluid’s insulating characteristics and improve dimensional accuracy, alumina, copper, and silicon are added [19]. Nanoparticles in the dielectric medium form a conductive bridge between the tool and workpiece at an appropriate voltage. These powder nanoparticles formed conductive chains under the electrode’s spark gap through scattering [20]. Once the frequency of sparking per unit of time grows, the workpiece material is removed quickly [21]. Adding powder lengthens the plasma channel, which raises spark density. Sparks frequently degrade the substance as a result. The workpiece’s perfect surface smoothness is the result of this consistent sparking [22]. Micro/nano powder in the dielectric fluid causes agglomeration that obstructs EDM machining and spark passage [23,24]. EDM agglomeration reduces sparking, changing output results. The addition of surfactants lessens the surface tension and migration of additives in the dielectric medium [25]. A surfactant is a surface-active chemical that is used to address the above issues [26]. Surfactants reduce nano-powder surface tension and scattering during EDM in the dielectric medium, increasing dispersion and conductivity. Resolving the issue improves dimensional accuracy [27]. Surfactants, including emulsifiers, improve geometric accuracy in dielectric fluid [28].

Materials used in EDM are prone to dimensional errors. Researchers are making progress in resolving the issue by using surfactants and metallic particles in dielectric fluid during EDM. This issue was resolved in earlier studies using electrodes that had been cryogenically treated (CT) against the base material during EDM. The wear, hardness, strength, toughness, and electrical properties of electrodes are enhanced by cryogenic treatment, which also enhances dimensional accuracy [29,30]. Ozdemir [31] found that a shallow CT at −84 °C increased the toughness, hardness, and wear of Cr-Fe compared to low-carbon cast steel. Senthilkumar and Rajendran [32] found that shallow cold working improved En 19 steel’s wear properties by 114% and deep cold working by 214%.

Tiwary et al. [33] investigated the Ti-6Al-4V alloy’s response to the brass electrode passing through various dielectric mediums. The authors came to the conclusion that at very low values of peak current, pure deionized water provided superior dimensional accuracy. However, the peak current, Cu mixed with deionized water dielectric, provided improved dimensional accuracy at the higher peak value. In order to explore the OC parameter following the machining of the aforementioned base material, Ahmed et al. [34] investigated the impact of several electrode materials on the titanium alloy. The authors concluded that each electrode displayed a unique pattern of OC performance. But if the discharge current was increased while the pulse time ratio was decreased, the graphite electrode produced the least OC magnitude. By pitting the various electrode materials against the EN 31 base material in the EDM oil as a dielectric medium, Singh et al. [35] evaluated the performance of EDM in terms of diametrical overcut. In comparison to Cu-W and brass electrodes, the scientists discovered that Cu and aluminum offered the lowest magnitude of OC against the mentioned base material. Sivakumar and Gandhinathan [36] examined the EDM’s machining capabilities in terms of OC when milling Ti-6Al-4V in comparison to various electrode materials. The scientists discovered that throughout the EDM process, the pulse on time and discharge current had the biggest effects on the OC size.

In terms of dimensional accuracy, Singh et al. [37] investigated the effect of the Cu electrode on the mild steel base material during EDM operation. The authors came to the conclusion that low discharge current and low pulse duration have a substantial impact on the output parameter, which results in improved dimensional accuracy. In order to assess the dimensional accuracy of CNC die-sinking EDM, Kumar et al. [3] examined the effect of the Cu electrode on P91 steel as the basic material in the presence of commercial-grade dielectric. The authors went on to explain that lower magnitudes of input parameters are taken into account in order to achieve a low OC magnitude. When using EN24 steel as the foundation material during electric discharge machining to evaluate OC, EWR, and SR, Grewal and Dhiman [38] compared the CT Cu electrode with the NT electrodes. According to the authors, OC magnitude is reduced by 9% with cryogenic treatment in comparison to simple electrodes. Bhaumik and Maity [39] assessed the radial OC, recast layer, surface roughness, and surface crack density during machining the Ti-5Al-2.5Sn titanium alloy in comparison to the Cu, brass, and zinc electrodes. The Cu electrode reportedly generated radial OC with the lowest magnitude, followed by brass and zinc at a lower peak current, according to the authors. Using a brass electrode and kerosene oil to cut the difficult-to-cut titanium superalloy, Pradhan et al. [40] investigated the dimensional precision of EDM. The key factors affecting the dimensional accuracy of the machined specimen were peak current and pulse on time. Kibria et al. [41] studied the effect of tungsten electrodes on the Ti-6Al-4V base material in the presence of simple and powdered dielectrics of deionized water and kerosene on EDM MRR, TWR, and OC. B4C powder in deionized water at a low discharge current on a tungsten electrode produced better dimensional precision than kerosene dielectric. Singh et al. [42] used the Taguchi technique to analyze EDM’s radial OC advantage over H-13 steel utilizing CT Cu and NT Cu electrodes. CT Cu electrode, 5A peak current, 5 μs pulse on time, and a 50 V discharge gap were found to minimize radial OC.

In order to achieve the best OC and EWR for the EDM of Inconel 600, Ishfaq et al. [43] employed five different biodegradable dielectrics with the addition of Cu powder. The authors discovered that the least amount of OC was attained in sunflower oil at the lowest value of Cu powder, but the smallest amount of EWR was obtained in the presence of amla oil at the lowest concentration of Cu powder. By using the different input factors during the micromachining,Das et al. [44] were able to determine the OC of Inconel 718. When compared to the other process parameters, the authors found that current had the biggest influence. Asif et al. [45] used surfactant-added biodegradable dielectrics for the EDM of Ti6Al4V alloy for biomedical applications to evaluate MRR, OC, TWR, and SR (Ra, Rz). The authors found an improvement of 41.7%, 80.3%, 75.3%, 55.3%, and 47.4%, respectively, with the addition of surfactant. Chaudhari et al. [46] machined the nickel-titanium shape memory alloy through wire electric discharge machining (WEDM) by engaging distinct process parameters. The authors revealed that at the optimal settings of process parameters, MRR was high, and SR was low. Moreover, the surface cracks, globules, and micropores were significantly reduced at the optimal parametric settings.

Pereira et al. [47] used the cryogenic cooling technique and minimum lubrication for the turning of AISI 304 steel. The authors revealed that with the cryogenic treatment, the tool life was improved by 50% and the cutting speed was enhanced by 30% compared to dry machining techniques. In another study, Pereira et al. [48] again used the cryogenic method for the machining of Inconel 718 with CO_2_ as an internal coolant. The results of this study revealed that cryo-milling Inconel 718 with the CO_2_ coolant increased the tool life by 57% in comparison to the emulsion coolant. In another study, Pereira et al. [49] reported that CO_2_ cryogenic turning increased the tool life by 60% by comparing the performance of two distinct inserts during the hard turning process. In addition to that, the authors also measured the superficial roughness and microstructure and found that positive inserts gave the best results. Sliusarenko et al. [50] suggested a new probe optimization method for (2 + 3)-axis inspection machines by moving the stylus at an inclined angle, by moving the stylus in a globally collision-free state, and by setting the stylus at a constant position. This optimized method compared the 3-axis and 5-axis optimization strategies and found that optimization results obtained by setting the stylus at a constant position were accurate compared to the results of the 3-axis and 5-axis approaches.

It is significant to remember that the EDM process is extremely complicated, non-linear, and controlled by the interaction of input factors. It can require some time and effort to develop a first-principle model for the process to forecast the profiles of OC under the modification of process control variables. The first-principle models could also be more reliable but are also fairly expensive to develop. To address the practical shortcomings of first principle models, another promising data-driven modeling paradigm incorporating a sophisticated and effective modeling method has evolved. An effective function approximation approach is the artificial neural network (ANN), which can create non-linear interactions between the process’ interactive input space. Additionally, the approach has shown remarkable performance in modeling systems at all scales, from atomic to enterprise [51,52].

Based on the literature cited above, it is clear that different possibilities have been explored for reducing the geometric inaccuracy of machined profiles in EDM. However, there is still a need to tune the electro-erosion process to improve control over the sparking phenomenon and have dimensionally consistent profiles. Specifically, this aspect is worth investigating as far as the formation of micro-impressions on IN617 is concerned. Therefore, the potentiality of the CT electrodes has been deeply examined for improving the control over sparking so that dimensional error may be minimized. The standard dielectric, i.e., kerosene, has also been modified in this context with the addition of Span and Tween-based surfactants to reduce the overcut magnitude. It is pertinent to mention that these customized kerosene-based dielectrics have never been explored from the perspective of geometric accuracy in the EDM of IN617. Moreover, EDM is a costly process, so it is very difficult for the industry to carry out a number of trials to make an appropriate selection of the control variables to produce accurate micro impressions. It is deemed necessary to effectively model this process to alleviate the need for experimentation. Considering the nonlinear characteristics of overcut in response to variation in input variables, an artificial neural network (ANN) approach is adopted for process modeling. The ANN technique efficiently maps input and output variables to predict the complex function space with reasonable accuracy. The development of an ANN-based model is another novel aspect of the study that eliminates the need for extensive experimentation and reduces cost. The experiment was run using a full factorial design. Through the use of statistical techniques and optical microscopy, the results are thoroughly examined. The science behind affecting dimensional accuracy has also been explained using scanning electron microscopy.

## 2. Materials and Methods

The supremacy of EDM is determined by the CT and NT electrodes against the Ni-based superalloy under the five kinds of modified dielectrics. Overcut (OC) or dimensional inaccuracy has to be taken as the single most important output response parameter. As mentioned earlier, IN617 has to be used as the base material; therefore, its chemical composition is evaluated by optical microscopy. Table 1 elaborates on the most significant workpiece characteristics [4]. The dimensions of the rectangular workpiece, in this case, are 60 mm × 60 mm × 5 mm.

**Figure 1:**
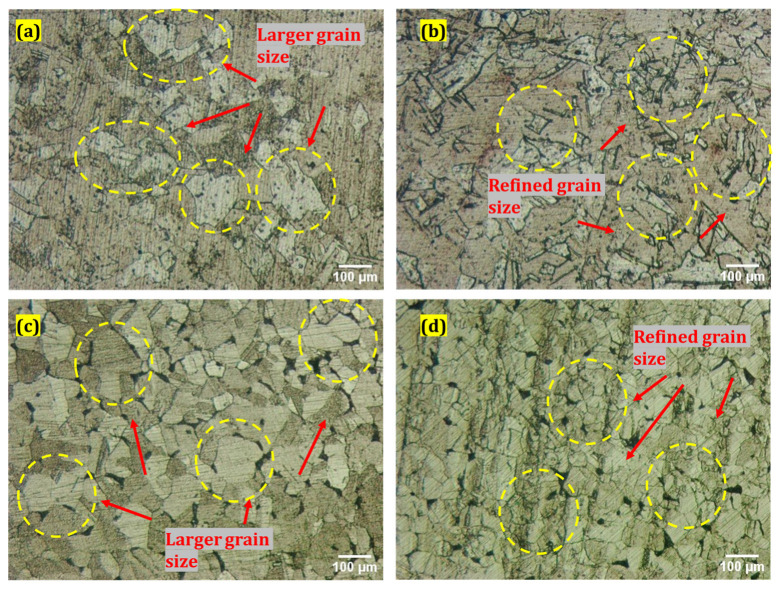
Microscopic images of (**a**) NT Cu electrode; (**b**) CT Cu electrode; (**c**) NT brass electrode; (**d**) CT brass electrode.

There are two types of electrodes that have been used in this study, i.e., copper (Cu) and brass, and both have an equal diameter of nine-millimeters. The performance of Cu and brass electrodes without cryogenic treatment and with cryogenic treatment has been investigated to elaborate the output parameter. The cryogenic operation was performed by exposing the electrodes to liquid nitrogen at −185 °C in the nitrogen container. The cryogenic procedure took 24 h at this temperature. Figure 1a–d indicates the microstructural grain refinement comparison of untreated and CT Cu and brass electrodes. However, the authors have calculated the average reduction in grain size using “ImageJ 1.54f” software. The optical microscopic images have been imported into the software, and measurements of grain sizes have been made at twenty-five grains. Then the average value is recorded. The same procedure is used for both treated and non-treated electrodes. The measurement of a non-treated electrode is made to have a reference value for the sake of comparison. The findings of this assessment indicate that the cryogenic treatment has notably reduced the grain size. In the case of Cu, an average reduction of 58.64% in grain size is noted, whereas for brass, a decrease of 50.62% in grain size is realized. Kerosene oil was used in this study as a dielectric. In addition to that, four different types of surfactants were also used at 6% concentration; a total of five unalike dielectric fluids were employed with the two kinds of CT and NT electrodes. Kerosene oil, kerosene oil in addition to Span 20 (Kerosene-S-20), kerosene oil in addition to Span 80 (Kerosene-S-80), kerosene oil in addition to Tween 20 (Kerosene-T-20), and kerosene oil in addition to Tween 80 (Kerosene-T-80) have been studied against the Cu and brass electrodes. Table 2 lists the major characteristics of the selected surfactants. The machine parameters were decided based on the results of the studies and are shown in Table 3 and Table 4. However, the physical properties of the used electrodes and the kerosene dielectric have been elaborated in Table 5 and Table 6.

**Figure 2:**
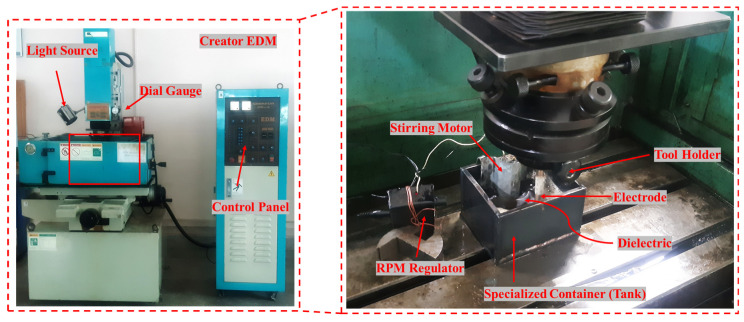
EDM working setup.

To obtain the best parametric settings where the minimum OC can be achieved, primary experiments were carried out. In the initial trials, those parameters that gave greater dimensional accuracy were chosen for the final experimentation of the workpiece against the CT and NT electrodes. In addition to that, the selection of these final parameters assisted in getting greater dimensional accuracy as well as reducing the cost of over-processing or burning the tool and workpiece during the EDM operation. Identifying the surfactant content in the kerosene oil dielectric was another crucial step. A starting point was taken from the literature, but the ultimate decision was based on preliminary experimental tests against the stated selection criteria. According to preliminary findings, 6% surfactant concentration is enough. To combine surfactant and kerosene oil, a motorized stirrer tank was constructed. Throughout the experiment, the stirrer maintains the surfactant and dielectric blended. An EDM (Model: RJ230) utilized for the experiment is seen in Figure 2.

An experimental design with a full factorial setup was used for the inquiry. The input parameters are summarized with levels in Table 3. Each test run included machining to a depth of cut of 0.3 mm. According to the experimental design, a total of 20 trials were completed. While the first ten trials employed CT electrodes, the latter ten used electrodes that were not CT. The output parameter was assessed once the experiment had been completed successfully. OC is the measurement of the average difference between the machined profile and the actual diameter of the electrode.

**Figure 3:**
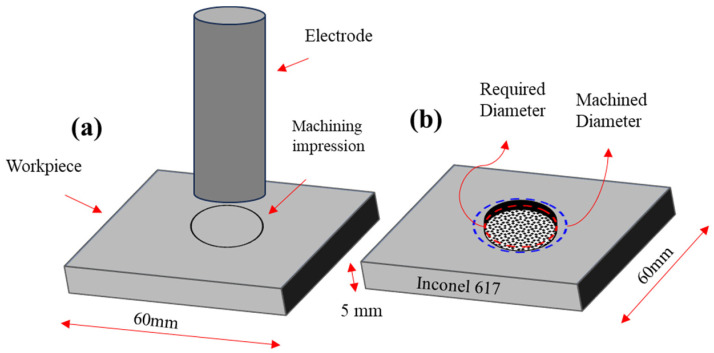
Schematic for the measurement of Overcut.

A coordinate measuring machine (CMM) is employed to measure the dimensional accuracy of the machined specimens after the EDM. Thereof, OCs were measured with Equation (1).

(1) $$Overcut=\frac{D_{m}-D_{t}}{2}$$

where *Dm* is the actual machined surface and *Dt* is the electrode diameter for the EDM of Ni-based superalloy. A schematic diagram for the measurement of dimensional accuracy is illustrated in Figure 3a,b. In Figure 3a, the workpiece and electrode materials have been presented, while in Figure 3b, the machining impression made during the EDM process has been elaborated. Bar charts were used to assess the data that had been gathered. The results and discussions section uses microscopic images to explain the over-machined specimen. The results are thoroughly explained, taking into account the physical processes involved in IN617 EDM. The appropriate selection of dielectric and electrode will be recommended for a smaller value of OC.

The causal input factors and the dimensional inaccuracy (OC) measured throughout the EDM process interact in non-linear and complex ways. It is not easy to model the intricate process’s output space, which is based on its causal input factors. The functional mapping between the input and output variables can be successfully developed using an artificial neural network, also referred to as a multilayer perceptron. The technique works well even on poorly specified systems and can efficiently mine the non-linear and complicated interactions in the hyperdimensional input space. The published material contains general information about the algorithm [55].

By adding just the correct degree of complexity to the model’s design and determining the ideal values for the network’s related parameters, the optimal performance of the ANN algorithm may be ensured. The complexity to be used to fit the curve across the data points is controlled by the number of neurons in the hidden layer of the ANN. A model’s capacity to generalize is significantly hampered by overly complex additions that may overfit the data curve and introduce noise. Similarly, a model’s prediction error is quite high if there are too few neurons, which may lead to a poor degree of fit across the data [56]. To overcome the difficulties described previously and create a model with great prediction capabilities, the number of neurons in the hidden layer of the ANN model must be appropriately chosen [57]. In general, the hidden layer’s number of neurons varies from 1× to 2.5× that of the input variables. The best-performing network can be chosen based on the comparative performance of the ANN models built upon the various numbers of hidden layer neurons [58]. The performance measurement parameters are included in a performance matrix that was created specifically for this activity. The root-mean-square error (*RMSE*), coefficient of determination (*R*^2^), and mean absolute error (MAE) are exceptional measurements to assess how well machine learning models train [59]. The mathematical expressions of *R*^2^, MAE, and *RMSE* are given as follows:

(2) $$R^{2}=1-\frac{{\sum_{i}^{N}(y_{i}-{\hat{y}}_{i})}^{2}}{{\sum_{i}^{N}(y_{i}-{\bar{y}}_{i})}^{2}}$$

(3) $$MAE=\frac{1}{n}\sum_{i=1}^{n}\left|y_{i}-{\hat{y}}_{i}\right|$$

(4) $$RMSE=\sqrt{\frac{1}{N}\sum_{i=1}^{N}{\left({\hat{y}}_{i}-y_{i}\right)}^{2}}$$

where, $y_{i}$ is the actual value of the output variables, whereas ${\hat{y}}_{i}$ is the model-predicted value of the output variable; ${\bar{y}}_{i}$ is the mean of $y_{i}$ and i = 1,2, 3…, *N* equal to the total number of observations. $R^{2}$ is a measure of accuracy and varies from zero (poor prediction performance) to one (perfect degree of fit), MAE measures the absolute error, whereas *RMSE* measures the error about the actual and model-predicted responses.

## 3. Results and Discussion

### 3.1. Experiments

**Figure 4:**
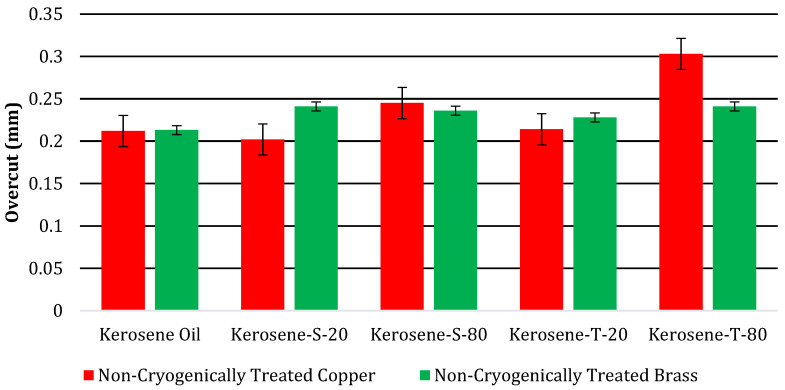
Comparison of overcut (mm) of NT electrodes.

The results of the OC magnitudes due to NT and CT electrodes of Cu and brass are displayed in Table 7. These results were obtained for five different modified dielectrics of kerosene. Moreover, to see the general behavior of these values, a bar chart of OC magnitudes due to NT electrodes is also drawn, as shown in Figure 4.

**Figure 5:**
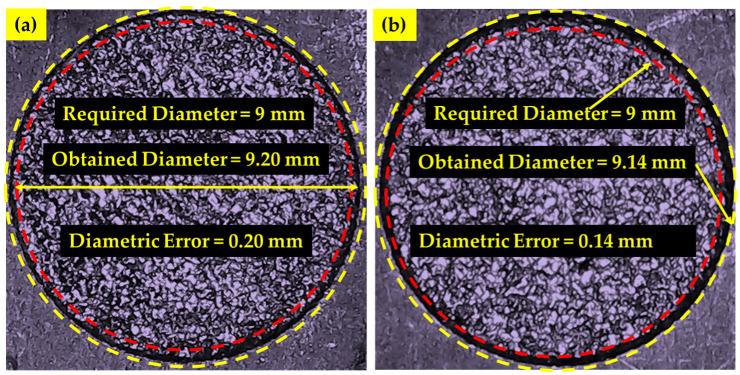
Diametric errors of machined surfaces in kerosene oil dielectric using; (**a**) NT electrodes; (**b**) CT electrodes.

**Figure 6:**
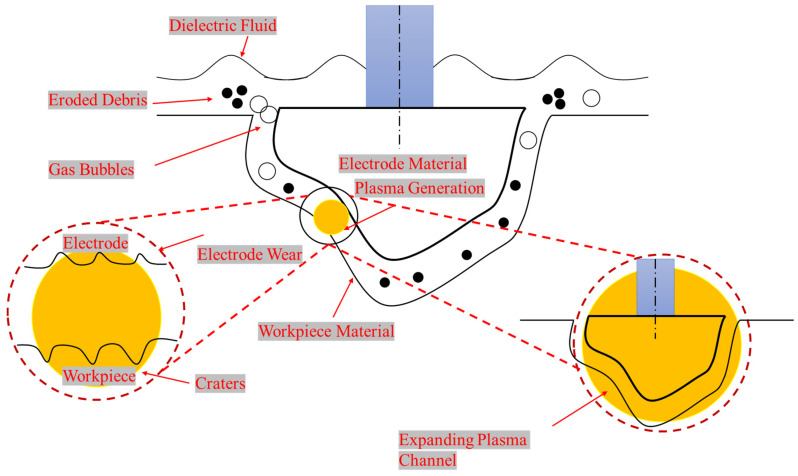
Schematic of dimensional inaccuracy due to side spark.

Figure 4 displays the findings about the OC magnitude caused by NT Cu and brass electrodes. It has been found that the NT Cu electrode produced the lowest OC magnitude (0.212 mm) during the EDM of superalloy. The discharge heat generated during the pulse duration is related to the smallest OC magnitude. The erosion of the base material increases with increasing discharge heat, and vice versa. As can be seen in Figure 5a, this resulted in less melting and vapourization occurring during the spark duration, which increases dimensional accuracy. The NT brass electrode provides the highest value of OC (0.213 mm), which accounts for the EDM’s dominance. The higher value of OC is a result of side sparking that happened when the NT brass electrode was engaged during the EDM operation, as shown in Figure 5b. Figure 6 depicts the schematic phenomena of side sparking, which leads to the degradation of the excess material beyond the electrode’s dimensions. The eroded material from the workpiece moved close to the corners of the electrode in the presence of dielectric, and these eroded particles then acted as the bridge between the tool and workpiece, which is why this site sparking is caused by the poor flushing of the eroded material. Every undesirable erosion in appliances, including this side sparking that removes excess and unneeded material, is disruptive [60].

**Figure 7:**
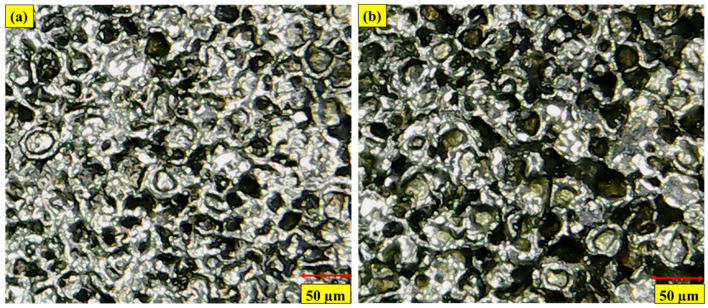
Micrographs of machined surfaces in Kerosene-S-20 modified dielectric using; (**a**) NT Cu electrodes; (**b**) NT brass electrodes.

In surfactant-added dielectric (Kerosne-S-20), NT Cu electrode machining proficiency was explored. The NT Cu electrode gave the lowest value of OC (0.202 mm) in the presence of S-20, which is 4.72% better than the magnitude of OC it gave in kerosene oil. This is because S-20 in the kerosene lowers surface tension and prevents erratic sparking from the Cu electrode. This prevents side sparking and improves dimensional accuracy. Figure 7a shows the Ni-based superalloy’s shallow craters under a microscope. In Kerosene-S-20’s dielectric, EDM’s NT brass electrode machining performance was assessed. NT brass electrodes had the highest OC magnitude (0.241 mm), 11.6% higher than kerosene oil dielectric. The higher magnitude of OC in the Kerosene-S-20 modified dielectric is due to the electrical conductivity of brass (16 × 10^6^ S/m) through the dielectric, which lowers its surface tension and increases radial sparks. Due to side sparking and material erosion, dimensional inaccuracies increased. Figure 7b shows IN617’s deeper craters. Brass electrode unevenness and radial sparking cause these deeper craters.

**Figure 8:**
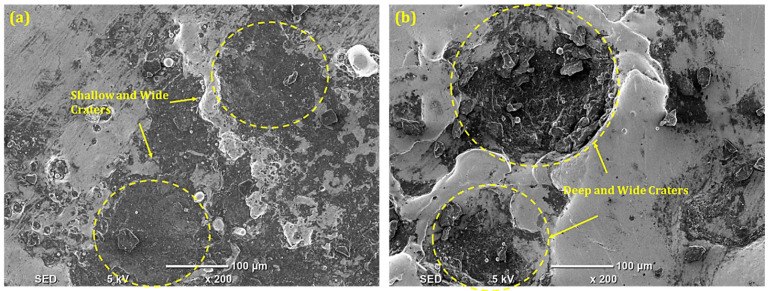
SEM of machined surface in Kerosene-S-80 modified dielectric using (**a**) NT brass electrode; (**b**) NT Cu electrode.

During the machining of IN617, the machining capability of EDM, due to the representative of spans, S-80, has been examined. It has been noted that when machining a Ni-based superalloy, NT brass electrodes produced the smallest OC magnitude (0.236 mm) in the modified Kerosene-S-80 dielectric. The aforementioned value is 2.10% better than the magnitude of the OC obtained in the presence of a modified Kerosene-S-20 dielectric. The greater flash point of S-80 (186.2 °C) is related to the lower and improved magnitude of OC caused by the NT brass electrode. The volatility and flammability will decrease the higher the dielectric’s flash point value. Because of this, the addition of S-80 to the kerosene dielectric lowers its higher flammability, increasing the dimensional accuracy of the modified kerosene-S-80 dielectric. Figure 8a of the SEM depicts shallow and small craters on the surface of IN617, where less radial spark favors less surface degradation and improves dimensional precision. Using a NT Cu electrode and a modified Kerosene-S-80 dielectric, the cutting power of EDM has been assessed. S-80 in kerosene and NT Cu electrodes gave the largest dimensional error (0.245 mm). This dimensional error is 17.6% larger than the OC’s magnitude obtained using the identical electrode material and S-20. The higher electrical conductivity of Cu (59 × 10^6^ S/m) and the decreased surface tension of kerosene brought on by the addition of S-80 are the causes of the larger dimensional inaccuracy. The aforementioned two characteristics of the electrode and surfactant combine to increase conductivity and cause radial sparking, which increases dimensional accuracy. Deep and wide craters on the EDMed surface, shown in Figure 8b, indicate substantial levels of material melting and evaporation. As a result, radial sparks also degrade the material past the boundaries of the electrodes and amplify dimensional errors.

The effectiveness of the EDM procedure has also been assessed using tweens, specifically T-20 in kerosene oil and NT Cu and brass electrodes. It was discovered that, the modified Kerosene-T-20 dielectric, the NT Cu electrode produced the OC with the least magnitude (0.214 mm). When compared to the magnitude of the OC produced in the modified dielectric made with kerosene-S-80, the dimensional inaccuracy acquired in the presence of kerosene-T-20 is 12.7% better. The NT Cu electrode in the kerosene oil results in lower and enhanced values of dimensional accuracy, which are caused by the high HLB (16.7) of T-20. The surface tension of the dielectric decreases with increasing HLB, preventing the agglomeration that leads to side sparking. When Ni-based superalloy is used in the modified Kerosene-T-20 dielectric against the NT Cu electrode, more dimensional precision is attained. Similar to this, the NT brass electrode used to investigate the superiority of EDM produced the highest value of OC when used with a modified Kerosene-T-20 dielectric. When compared to the OC’s magnitude acquired in the presence of a modified dielectric made from kerosene-S-80, the dimensional accuracy reached in kerosene-T-20 is 3.40% better. The preceding findings discussed in the above section are related to the explanation for the increased value of OC brought on by the NT brass electrodes.

**Figure 9:**
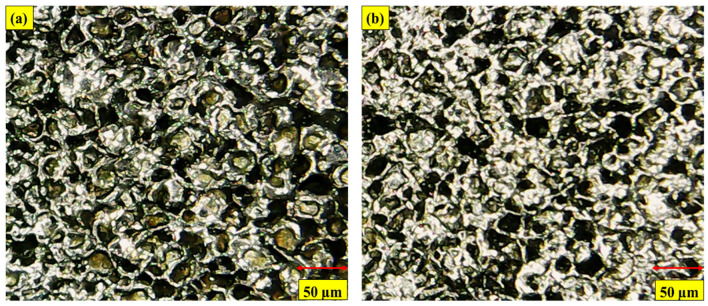
Micrographs of machined surfaces in Kerosene-T-80 modified dielectric using; (**a**) NT Cu electrodes; (**b**) NT brass electrodes.

**Figure 10:**
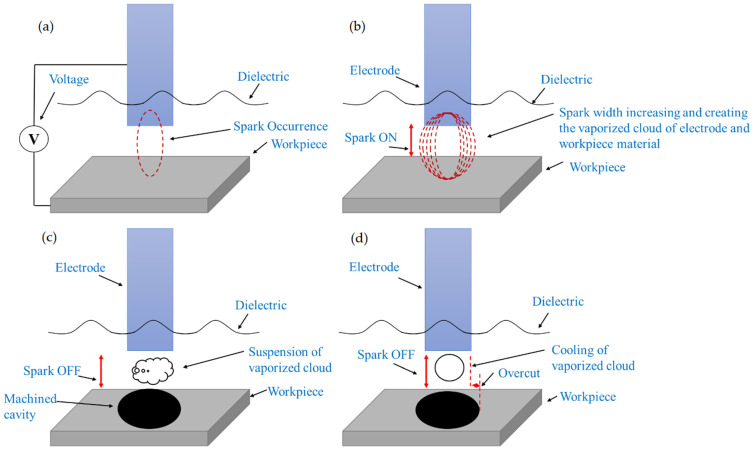
Overcut schematic of machined surface in Kerosene-T-80 modified dielectric using NT Cu electrode.

T-80, a tween integrated with kerosene dielectric, gave the lowest OC (0.241 mm) due to the NT brass electrode during EDM of a difficult-to-machine superalloy. T-80 and a NT brass electrode raise OC magnitude by 5.4% compared to Kerosene-T-20 modified dielectric medium with the same electrode. The NT brass electrode increased OC since T-80 had a lower HLB (15) than T-20, which increased dimensional inaccuracy. Figure 9a shows deep craters caused by brass electrode grain sizes. Large grain sizes erode material beyond electrode dimensions. The EDM technique is superior since the NT Cu electrode in Kerosene-T-80 had the highest dimensional error (0.303 mm). OC is 29.4% higher than with Kerosene-T-20 modified dielectric. The NT Cu electrode in T-80 may cause poor dimensional accuracy for two reasons. Cu has a greater electrical conductivity (59 × 10^6^ S/m), and T-80 has the second-greatest HLB (15). The OC value is increased by the two traits. Figure 10a–d shows that the NT Cu electrode had the highest OC value with T-80. Figure 10a illustrates that the spark is initiating while the Figure 10b shows that radial sparking is expanding and with the pulse-on-time the sparking width has been increased and the highest dimensional error is achieved. Figure 10c shows that a rising spark width in spark duration melts and vaporizes the workpiece material, generating an OC magnitude during EDM. Figure 10d indicates the machined EDMed surface with dimensional inaccuracy. Figure 9b shows the Ni-based superalloy’s shallow craters under a microscope. Regular sparking is indicated by shallow craters.

**Figure 11:**
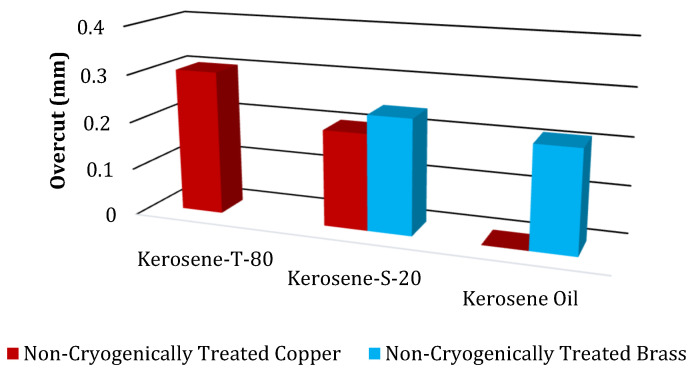
Comparison of highest and lowest value of OC due to NT electrodes.

**Figure 12:**
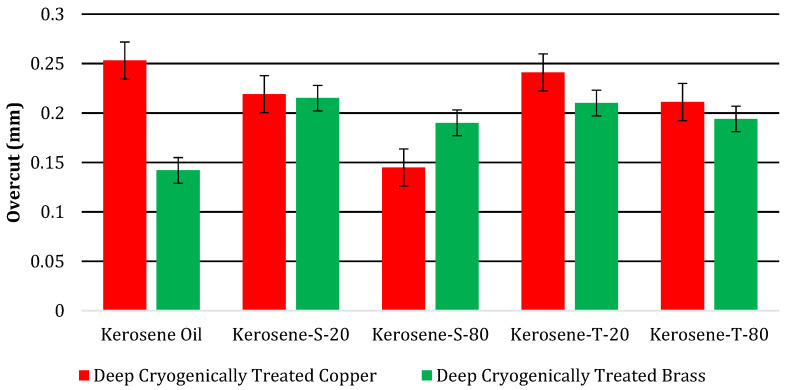
Comparison of Overcut (mm) of different CT electrodes.

The findings of NT electrodes for machining hard-to-machine Ni-based superalloys are as follows: EDM efficiency has been determined by the NT Cu electrode, which yields the lowest OC (0.202 mm) in the Kerosene-S-20 modified dielectric medium. OC is 4.72% better than in kerosene oil with the same electrode material. Kerosene oil reduces OC to 0.213 mm with a NT brass electrode. Figure 11 compares the maximum and minimum OC values of NT Cu and brass electrodes in different dielectric mediums. Figure 12 shows CT Cu and brass electrode OC magnitude bar plots. IN617 is machined with five modified kerosene oil dielectrics.

**Figure 13:**
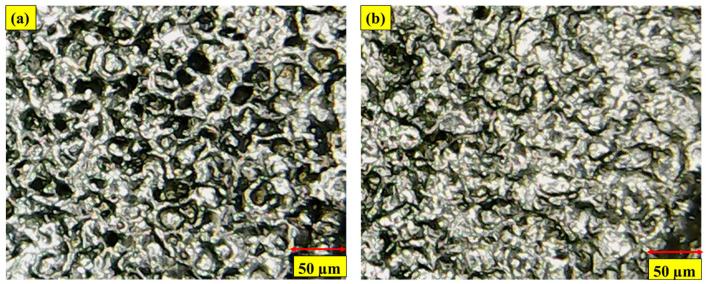
Micrographs of machined surfaces in Kerosene dielectric using; (**a**) CT Cu electrodes; (**b**) CT brass electrodes.

**Figure 14:**
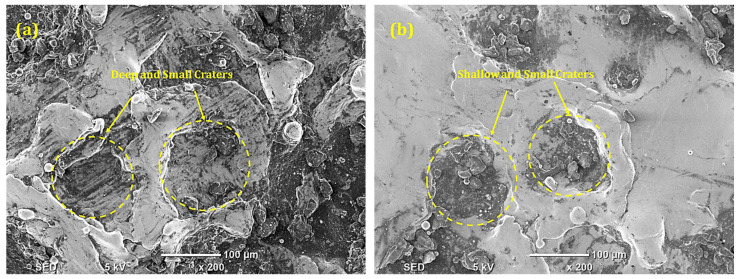
SEM of machined surface in Kerosene-S-20 modified dielectric using (**a**) CT Cu electrode; (**b**) CT brass electrode.

In kerosene oil, CT Cu electrodes gave the highest value of OC (0.253 mm) for EDM cutting. Cryogenic treatment enhanced the thermal conductivity of the CT Cu electrode, causing greater material degradation at the edges and a higher OC value. Figure 13a shows a structural refinement of the electrode due to which deep craters are formed on the machined profile’s surface. Cryogenic treatment also reduces the radial sparking of the electrode. Figure 12 shows that the CT brass electrode EDM is superior. The CT brass electrode gave the lowest OC magnitude (0.142 mm) in kerosene oil during superalloy machining. Cryogenic treatment on the brass electrode increases its grain structure, wear, toughness, and hardness, which lowers OC. Cryogenic treatment improves the uneven sparking caused by NT brass, removing uniform sparking from the electrode and improving dimensional accuracy, as illustrated in Figure 13b. Figure 14a,b show CT Cu and brass electrode craters in machined profiles. A better surface polish indicates shallow craters.

The representatives of spans (S-20) were used to examine the EDM operation of IN617 in the dielectric. CT brass electrodes had the lowest OC magnitude (0.215 mm) in modified dielectric Kerosene-S-20. OC magnitude is 34.0% higher than in the presence of kerosene oil during Ni-based superalloy EDM. S-20 lowers dielectric medium surface tension, and cryogenic treatment improves electrode conductivity, increasing the OC value. EDM was tested with a CT Cu electrode against the IN617. The CT Cu electrode had the largest OC magnitude (0.253 mm) in the Kerosene-S-20 modified dielectric medium. Kerosene-S-20 dielectric has 13.4% better dimensional accuracy than kerosene oil. S-20 with kerosene oil lowers the OC value. S-20 prevents dielectric agglomeration, and cryogenic treatment improves electrode grain structure and decreases side sparking, improving dimensional accuracy.

The CT Cu electrode has the lowest OC (0.149 mm) in the Kerosene-S-80 modified dielectric. The CT Cu electrode improves dimensional accuracy by 32.0% compared to the S-20 dielectric. Cryogenic treatment on the Cu electrode and a greater flash point of S-80 (186.2 °C) decrease OC. Volatility and flammability decrease with the increment of the dielectric flash point. The S-80 additive reduces the flammability of the kerosene dielectric, and the consistent grain structure reduces erratic sparking, resulting in increased dimensional accuracy. S-80 showed EDM’s machining performance with CT brass electrodes having the maximum OC (0.190 mm). OC is 11.6% improved compared with Kerosene-S-20 modified dielectric. Cryogenic treatment increases brass’s electrical conductivity (16 × 10^6^ S/m), and S-80 lowers kerosene’s surface tension, reducing dimensional inaccuracy. The electrode and surfactant combine to increase conductivity but decrease side sparking compared to S-20 with the treated brass electrode, resulting in improved dimensional precision.

**Figure 15:**
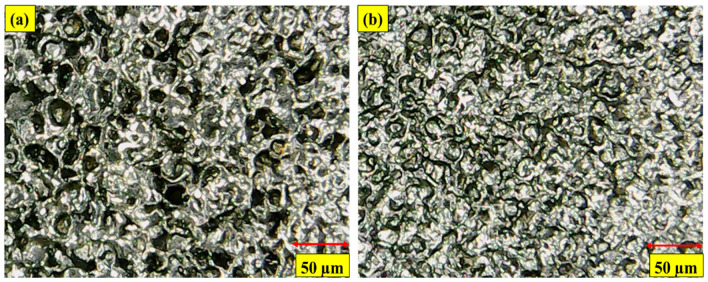
Micrographs of machined surfaces in Kerosene-T-20 modified dielectric using; (**a**) CT Cu electrodes; (**b**) CT brass electrodes.

The CT Cu electrode had the maximum OC (0.241 mm) in the Kerosene-T-20 modified dielectric. The CT Cu electrode improves dimensional accuracy by 38.1% over the S-80 dielectric. CT Cu electrodes in T-20 may have low dimensional accuracy for two reasons. The first reason is Cu’s electrical conductivity (59 × 10^6^ S/m) and T-20’s highest HLB (16.7). Side sparking melts the Ni-based superalloy and causes over-machining during pulse on time, which increases dimensional inaccuracy. Figure 15a shows shallow craters that remove significant material during sparking. EDM’s machining performance was assessed using T-20 in kerosene to cut IN617. CT brass electrodes had the lowest OC (0.210 mm) in Kerosene-T-20 modified dielectric. The OC magnitude is 9.5% rougher than with the S-80 modified dielectric of kerosene. T-20’s high HLB (16.7) explains the CT brass electrode’s lower and rougher OC readings in kerosene oil. Greater the HLB, lower the dielectric surface tension. Cryogenic treatment enhanced brass conductivity, resulting in poor dimensional accuracy when Ni-based superalloy is used against the CT brass electrode in the modified dielectric of Kerosene-T-20. Figure 15b shows the improved surface. Thus, the machined cavity’s boundaries had the same surface and less over-machining.

The CT brass electrode had the lowest OC magnitude (0.194 mm) in modified dielectric Kerosene-T-80. This OC magnitude is 7.6% better than in Kerosene-T-80 during Ni-based superalloy EDM. The CT brass electrode’s lower specific heat capacity (0.380 J/g °C) and T-80’s lower HLB (15) are lower than T-20’s increased OC. EDM was tested with a CT Cu electrode against the IN617. The CT Cu electrode had the maximum OC magnitude (0.211 mm) in the Kerosene-T-80 modified dielectric medium. Kerosene-S-20 dielectrics provide 12.4% improved dimensional accuracy compared to Kerosene-T-20. The above cryogenic sections show why the CT Cu electrode improves OC.

**Figure 16:**
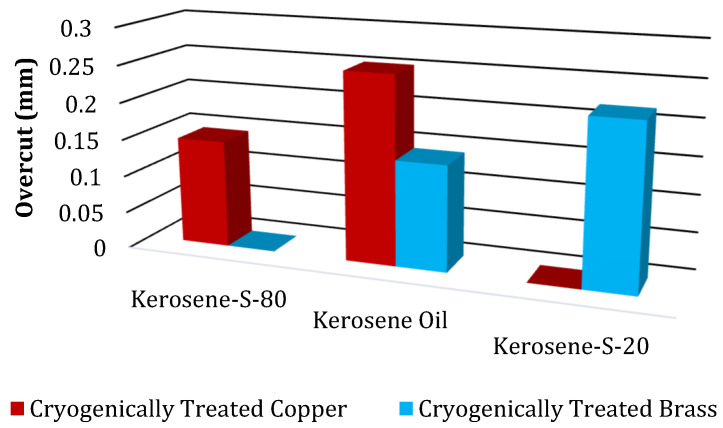
Comparison of maximum and minimum value of OC due to CT electrodes.

CT electrodes for hard-to-machine Ni-based superalloy machining has the following findings. The CT brass electrode has the lowest OC (0.142 mm) in kerosene oil. EDM efficiency has been measured by the CT Cu electrode, which yields the lowest OC (0.145 mm) in the Kerosene-S-80 modified dielectric medium. OC is 42.7% better than in kerosene oil with the same electrode material. Figure 16 compares the maximum and minimum OC values of CT Cu and brass electrodes in different dielectric mediums.

### 3.2. Development of Artificial Neural Network (ANN) for OC

Artificial neural networks (ANN) belong to the fastest-growing areas of Artificial Intelligence (AI) and have become a key component of many advanced AI systems. Flexibility, adaptability, and generalization capability are the most important ANN features. ANNs are capable of learning, adapting, and improving their performance based on available data and experience. This unique ability allows them to be applied to a wide range of tasks. ANNs are also excellent universal approximators as they can represent and learn any function, which is extremely useful in a wide range of applications [61]. Due to their ability to detect hidden patterns and relationships in data, artificial neural networks can generalize the learned information to new, previously unknown cases. This is particularly valuable as they can deal with incomplete or unknown data. Therefore, they are considered tools that may overcome the shortcomings of the time-consuming and expensive techniques of data handling, including the programmed computing approach and the experimental procedures.

This work develops a three-layered shallow artificial neural network (ANN) model to model OC. The neurocomputing approach is an effective way to split the data ratio into 0.8, 0.1, and 0.1 and is used for training, testing, and validation datasets, respectively. The activation function applied to the hidden and output layers is tangent sigmoidal and linear, respectively. The Levenberg–Marquart algorithm is applied to optimize the parametric values (weights and biases), and the sum-of-squared error is taken as the training function.

Two hyperparameters, namely the number of hidden layer neurons and learning rate, are extensively investigated to develop a well-predicting ANN model. The number of neurons is generally changed from 1× to 2.5× in the input layer, thus varying from 2 to 5 in this work. Furthermore, the learning rate is studied in the range of 1 × 10^−4^ to 1 × 10^−1^, typically reported in research studies.

**Figure 17:**
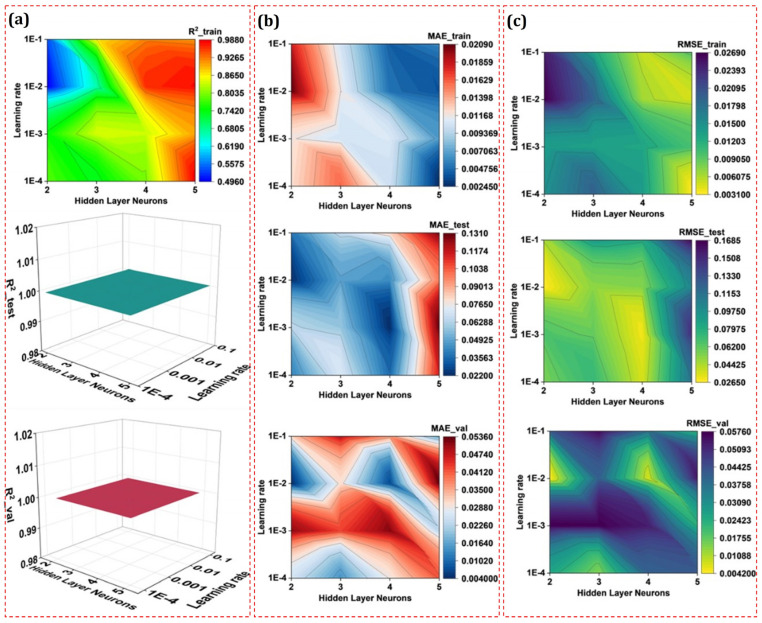
Development of ANN under various hidden layer neurons and learning rate to model OC. The modeling performance of the ANN is evaluated on (**a**) R^2^, (**b**) MAE, and (**c**) RMSE for training, testing, and validation datasets.

Figure 17 presents the modeling performance of ANN to develop a functional mapping between the input and output variables concerning the number of hidden layer neurons and learning rate. The modeling performance is measured by R^2^, MAE, and RMSE for the training, testing, and validation datasets. Referring to the modeling performance of the ANN in the training phase, the R^2^ value varied from 0.5 to 0.98. At the same time, R^2^ remains one for the testing and validation dataset, irrespective of the hidden layer neurons. When closely comparing the modeling performance of ANN on MAE and RMSE for hidden layer neurons and learning rate, there is a large variation in the error values in the training, testing, and validation datasets. However, it is found that the ANN model having four hidden layer neurons and a learning rate of 0.01 has comparatively improved values of performance matrices as follows: R^2^_train = 0.96, R^2^_test = 1, R^2^_val = 1, MAE_train = 0.005 mm, MAE_test = 0.043 mm, MAE_val = 0.007 mm, RMSE_train = 0.006 mm, RMSE_test = 0.053 mm, and RMSE_val = 0.008 mm. The comparative modeling performance of ANNs under three datasets enables the selection of a better ANN that possesses good prediction and generalization performance. Thus, the ANN model with four hidden layer neurons is selected to predict the OC based on input variables.

**Figure 18:**
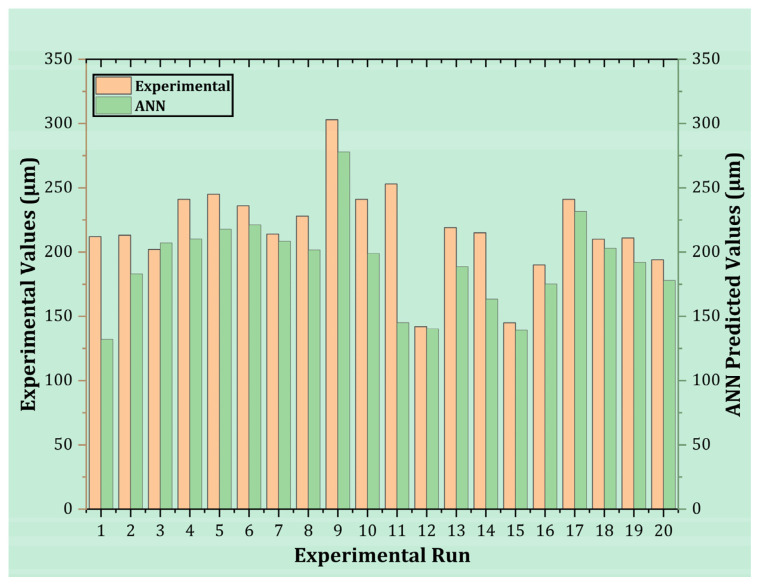
Comparison of experimental and ANN-predicted values of OC.

As it was mentioned previously, the ANN model is used to predict the values of OC, and the R^2^ of every run is greater than 0.96. Currently, if a comparison is developed between the experimental runs and the predicted values of ANN, then it can be seen that ANN performed exceptionally well in every run compared to the values of OC obtained in actual experimentation, as shown in Figure 18. Therefore, it is concluded that ANN is a better-performing model for estimating the values of output responses.

## 4. Conclusions

The machining efficiency of NT and CT electrodes constructed of copper and brass in terms of dimensional accuracy has been evaluated while cutting a Ni-based superalloy. The results are explained in terms of process physics and potential primary reasons. An artificial neural network (ANN) has also been developed to forecast the values of OC. The following conclusions are derived after a detailed examination of the behavior of NT and CT electrodes under various kerosene oil-modified dielectrics:

1. In various modified dielectrics, the ability of CT electrodes to machine gave greater dimensional accuracy on average by 13.5% compared to NT electrodes;
2. CT brass outperformed the treated electrodes and provided the lowest value of OC (0.142 mm). Compared to the average value provided by the total number of CT electrodes, the dimensional accuracy provided by the CT brass is 29.7% better/enhanced;
3. A comparison of the machining capabilities of CT electrodes in span and tween-added dielectrics revealed that span-based modified dielectrics provided an 8.7% reduced overcut;
4. When using a CT Cu electrode in pure kerosene oil, EDM achieves the lowest OC (0.145 mm) and the highest dimensional accuracy (31.6% better than pure kerosene oil in the NT situations);
5. The combination of a NT Cu electrode and the Kerosene-S-20 modified dielectric has yielded the lowest value of OC (0.202 mm). This OC value is 33.3% better than the best OC recorded using a NT Cu electrode and a Kerosene-T-80 modified dielectric;
6. It has been shown that tween-added dielectrics against NT electrodes perform 3.0% better than span-based modified dielectrics in machining ability. Tweens perform better than teenagers because their high flash points prevent them from catching fire easily;
7. In Kerosene-S-20 and kerosene oil-modified dielectrics, the lowest OC was achieved by proficient NT Cu and brass machining, with 0.202 mm and 0.213 mm, respectively. Kerosene-S-20’s OC value is said to be 4.72% better than kerosene oil’s OC value because of NT Cu;
8. The OC phenomenon is observed to possess nonlinear characteristics and is therefore modeled using the ANN approach. Extensive tuning of hyperparameters is carried out; thus, an ANN model with four hidden layer neurons and a learning rate of 0.01 has comparatively better modeling performance. The developed ANN model can accurately predict the OC value in the design input space.

In this research, the proficiency of cryogenic-treated and non-treated electrodes has been deeply examined under five dielectrics for improving the geometrical accuracy of machined profiles. The process has also been successfully modeled using the ANN approach. An optimized parametric combination has also been proposed. However, the work can be extended to formulate a physical mathematical model of the EDM process. The potential assessment of hybrid combinations of dielectrics is another aspect that can be explored. Powder-based additives can also be investigated for improving the dimensional accuracy of the machined profiles in EDM.

## Figures and Tables

**Table 1:** Chemical and physical attributes of IN617.

Element	% Content	Element	% Content	Properties	Values
C	0.05–0.15	Cu	0.5	Electrical Resistivity (µ Ωm)	1.22
S	0.015	Co	10.0–15.0	Melting Range (°C)	1332–1380
Cr	20.0–24.0	Mo	8.0–10.0	Specific Heat (J/kg°C)	419
Ti	0.6	Mn	1.0	Density (kg/m^3^)	8360
Ni	44.5	Al	0.8–1.5	-	-
Si	1.0	B	0.006	-	-
Fe	3.0	-	-	-	-

**Table 2:** Surfactant’s physical and chemical properties.

Properties	Chemical Formula	Molecular Weight (g/mol)	Density (g/cm^3^ at 25 °C)	Flashpoint (°C)	HLB Value
S-80	C_24_H_44_O_6_	428.60	1.068	186.2	4.6
S-20	C_18_H_34_O_6_	346.46	1.032	>110	8.6
T-80	C_64_H_124_O_26_	1309	1.08	148	15
T-20	C_58_H_114_O_26_	1227.54	1.095	>110	16.7

**Table 3:** Levels of process parameters.

Input Variable	Levels				
	1	2	3	4	5
Type of Dielectric	Pure Kerosene	Kerosene-S-20	Kerosene-S-80	Kerosene-T-20	Kerosene-T-80
Type of Surfactant	S-20	Span-80	Tween-20	Tween-80	-
Electrode Type	Copper	Brass	-	-	-
Treatment Type	NT	CT	-	-	-

**Table 4:** Constant Parameters.

Constant Factors (Units)	Values
Surfactant Concentration (Vol %)	6
Spark Voltage (Volts)	4
Current (Amperes)	10
Pulse off Time (µsec)	26
Pulse on Time (µsec)	100

**Table 5:** Attributes of the electrodes [53,54].

Properties	Melting Point (°C)	Electrical Conductivity (S/m)	Density (g/mm^3^)	Specific Heat Capacity (J/g °C)	Electrical Resistivity (Ω.m)
Copper	1083	59 × 10^6^	8.904 × 10^−3^	0.835	1.96 × 10^−8^
Brass	990	16 × 10^6^	8.55 × 10^−3^	0.38	4.7 × 10^−7^

**Table 6:** Properties of kerosene oil [4,55].

Properties	Kerosene Oil
Specific heat (kJ/kg K)	2.0
Dielectric constant	2.12
Density (gm/cm^3^) at 20 °C	0.8
Flashpoint (°C)	54
Thermal conductivity (W/mK)	0.12
Viscosity (cm^2^/s)	1.22
Breakdown Voltage (kV)	48

**Table 7:** OC values of NT and CT electrodes.

Sr. No	Modified Dielectric	Electrode Types	OC (mm)	Sr. No	Modified Dielectric	Electrode Types	OC (mm)
1	Kerosene	NT copper	0.212	11	Kerosene	CT copper	0.253
2		NT brass	0.213	12		CT brass	0.142
3	Kerosene-S-20	NT copper	0.202	13	Kerosene-S-20	CT copper	0.219
4		NT brass	0.241	14		CT brass	0.215
5	Kerosene-S-80	NT copper	0.245	15	Kerosene-S-80	CT copper	0.145
6		NT brass	0.236	16		CT brass	0.19
7	Kerosene-T-20	NT copper	0.214	17	Kerosene-T-20	CT copper	0.241
8		NT brass	0.228	18		CT brass	0.21
9	Kerosene-T-80	NT copper	0.303	19	Kerosene-T-80	CT copper	0.211
10		NT brass	0.241	20		CT brass	0.194

## Data Availability

Not applicable.

## References

[1] Sun Y., Gong Y., Wen X., Xin B., Yin G., Meng F., Tang B. (2022). Evaluation of Dimensional Accuracy and Surface Integrity of Cylindrical Array Microelectrodes and Cylindrical Array Microholes Machined by EDM. Archiv. Civ. Mech. Eng..

[2] Sahu D.R., Mandal A. (2020). Critical Analysis of Surface Integrity Parameters and Dimensional Accuracy in Powder-Mixed EDM. Mater. Manuf. Process..

[3] Kumar P., Dewangan S., Pandey C. (2020). Analysis of Surface Integrity and Dimensional Accuracy in EDM of P91 Steels. Mater. Today Proc..

[4] Ishfaq K., Waseem M.U., Sana M. (2022). Investigating Cryogenically Treated Electrodes’ Performance under Modified Dielectric(s) for EDM of Inconel(617). Mater. Manuf. Process..

[5] Li W., Guo Y.B. A Two-Parameter Method to Monitor and Characterize Tool Wear in End Milling Inconel 718. Proceedings of the ASME 2012 International Manufacturing Science and Engineering Conference, Notre Dame, IN, USA, 4 June 2012.

[6] Guo Y.B., Li W., Jawahir I.S. (2009). Surface Integrity Characterization and Prediction in Machining of Hardened and Difficult-to-Machine Alloys: A State-of-Art Research Review and Analysis. Mach. Sci. Technol..

[7] Sidhu S.S., Ablyaz T.R., Bains P.S., Muratov K.R., Shlykov E.S., Shiryaev V.V. (2021). Parametric Optimization of Electric Discharge Machining of Metal Matrix Composites Using Analytic Hierarchy Process. Micromachines.

[8] Shastri R.K., Mohanty C.P., Dash S., Gopal K.M.P., Annamalai A.R., Jen C.-P. (2022). Reviewing Performance Measures of the Die-Sinking Electrical Discharge Machining Process: Challenges and Future Scopes. Nanomaterials.

[9] Dilip D.G., Panda S., Mathew J. (2020). Characterization and Parametric Optimization of Micro-Hole Surfaces in Micro-EDM Drilling on Inconel 718 Superalloy Using Genetic Algorithm. Arab. J. Sci. Eng..

[10] Altin Karataş M., Biberci M.A. (2022). Statistical Analysis of WEDM Machining Parameters of Ti-6Al-4V Alloy Using Taguchi-Based Grey Relational Analysis and Artificial Neural Network. Exp. Tech..

[11] Gangil M., Pradhan M.K. (2017). Modeling and Optimization of Electrical Discharge Machining Process Using RSM: A Review. Mater. Today Proc..

[12] Kolli M., Kumar A. (2015). Effect of Dielectric Fluid with Surfactant and Graphite Powder on Electrical Discharge Machining of Titanium Alloy Using Taguchi Method. Eng. Sci. Technol. Int. J..

[13] Fassi I., Modica F. (2021). Editorial for the Special Issue on Micro-Electro Discharge Machining: Principles, Recent Advancements and Applications. Micromachines.

[14] Qudeiri J.E.A., Zaiout A., Mourad A.-H.I., Abidi M.H., Elkaseer A. (2020). Principles and Characteristics of Different EDM Processes in Machining Tool and Die Steels. Appli. Sci..

[15] Jafarian F. (2020). Electro Discharge Machining of Inconel 718 Alloy and Process Optimization. Mater. Manuf. Process..

[16] Singh K., Agarwal A.K., Yadav R. (2017). Effect of Dielectric Fluids Used on EDM Performance: A Review. IJETER.

[17] Bhattacharyya B., Doloi B. (2020). Machining Processes Utilizing Thermal Energy. Modern Machining Technology.

[18] Joshi A.Y., Joshi A.Y. (2019). A Systematic Review on Powder Mixed Electrical Discharge Machining. Heliyon.

[19] Zhao W.S., Meng Q.G., Wang Z.L. (2002). The Application of Research on Powder Mixed EDM in Rough Machining. J. Mater. Process. Technol..

[20] Kumar A., Maheshwari S., Sharma C., Beri N. (2011). Analysis of Machining Characteristics in Additive Mixed Electric Discharge Machining of Nickel-Based Super Alloy Inconel 718. Mater. Manufac. Process..

[21] Kumar A., Maheshwari S., Sharma C., Beri N. (2010). Research Developments in Additives Mixed Electrical Discharge Machining (AEDM): A State of Art Review. Mater. Manuf. Process..

[22] Jamadar M.U.M., Kavade M.V. (2014). Effect of Aluminium powder mixed EDM on machining characteristics of die steel (AISI D3). IJMPE.

[23] Dewan P.R., Kundu P.K., Phipon R. Powder Mixed Electric Discharge Machining—A Review. Proceedings of the 2nd International Conference on Mechanical Materials and Renewable Energy (Icmmre 2019).

[24] Al-Amin M., Abdul-Rani A.M., Rana M., Hastuty S., Danish M., Rubaiee S., Mahfouz A. (2022). bin Evaluation of Modified 316L Surface Properties through HAp Suspended EDM Process for Biomedical Application. Surf. Interfaces.

[25] Qazi M.J., Schlegel S.J., Backus E.H.G., Bonn M., Bonn D., Shahidzadeh N. (2020). Dynamic Surface Tension of Surfactants in the Presence of High Salt Concentrations. Langmuir.

[26] Dave N., Joshi T. (2017). A Concise Review on Surfactants and Its Significance. Int. J. Appl. Chem..

[27] Ilani M.A., Khoshnevisan M. (2021). Study of Surfactant Effects on Intermolecular Forces (IMF) in Powder-Mixed Electrical Discharge Machining (EDM) of Ti-6Al-4V. Int. J. Adv. Manuf. Technol..

[28] Bart J.C.J., Gucciardi E., Cavallaro S. (2013). Formulating Lubricating Oils. Biolubricants.

[29] Villarrazo N., Caneda S., Pereira O., Rodríguez A., López De Lacalle L.N. (2023). The Effects of Lubricooling Ecosustainable Techniques on Tool Wear in Carbon Steel Milling. Materials.

[30] Kumar S., Khedkar N.K., Jagtap B., Singh T.P. (2017). The Effects of Cryogenic Treatment on Cutting Tools. IOP Conf. Ser. Mater. Sci. Eng..

[31] Özdemir Z. (2021). Shallow Cryogenic Treatment (SCT) Effects on the Mechanical Properties of High Cr Cast Iron: Low-Carbon Cast Steel Bimetallic Casting. Inter Met..

[32] Senthilkumar D., Rajendran I. (2011). Influence of Shallow and Deep Cryogenic Treatment on Tribological Behavior of En 19 Steel. J. Iron Steel Res. Int..

[33] Tiwary A.P., Pradhan B.B., Bhattacharyya B. (2018). Investigation on the Effect of Dielectrics during Micro-Electro-Discharge Machining of Ti-6Al-4V. Int. J. Adv. Manuf. Technol..

[34] Ahmed N., Ishfaq K., Rafaqat M., Pervaiz S., Anwar S., Salah B. (2019). EDM of Ti-6Al-4V: Electrode and Polarity Selection for Minimum Tool Wear Rate and Overcut. Mater. Manufac. Process..

[35] Singh S., Maheshwari S., Pandey P.C. (2004). Some Investigations into the Electric Discharge Machining of Hardened Tool Steel Using Different Electrode Materials. J. Mater. Process. Technol..

[36] Sivakumar K.M., Gandhinathan R. (2013). Establishing Optimum Process Parameters for Machining Titanium Alloys (Ti6Al4V) in Spark Electric Discharge Machining. IJEAT.

[37] Singh B., Kumar P., Arora G., Kumar A. (2014). Analysis of Machining Parameters for Dimensional Accuracy in EDM Using Taguchi’s Doe. Int. J. Eng. Res..

[38] Grewal G.S., Dhiman D.P. (2019). Effect of Deep Cryogenic Treatment on Copper Electrode for Non-Traditional Electric Discharge Machining (EDM). Mech. Sci..

[39] Bhaumik M., Maity K. (2019). Effect of Electrode Materials on Different EDM Aspects of Titanium Alloy. Silicon.

[40] Pradhan B.B., Masanta M., Sarkar B.R., Bhattacharyya B. (2009). Investigation of Electro-Discharge Micro-Machining of Titanium Super Alloy. Int. J. Adv. Manuf. Technol..

[41] Kibria G., Sarkar B.R., Pradhan B.B., Bhattacharyya B. (2010). Comparative Study of Different Dielectrics for Micro-EDM Performance during Microhole Machining of Ti-6Al-4V Alloy. Int. J. Adv. Manuf. Technol..

[42] Singh P.B., Phull G.S., Puggal S. (2015). Study of Radial Overcut during EDM of H-13 Steel with Cryogenic Cooled Electrode Using Taguchi Method. Int. J. Mech. Eng. Robot. Res..

[43] Ishfaq K., Sana M., Rehman M., Anwar S., Alfaify A.Y., Zia A.W. (2023). Role of Biodegradable Dielectrics toward Tool Wear and Dimensional Accuracy in Cu-Mixed Die Sinking EDM of Inconel 600 for Sustainable Machining. J. Braz. Soc. Mech. Sci. Eng..

[44] Das A., Padhan S., Ranjan Das S. (2022). Analysis on Hole Overcut during Micro-EDM of Inconel 718. Mater. Today Proc..

[45] Asif N., Saleem M.Q., Farooq M.U. (2023). Performance Evaluation of Surfactant Mixed Dielectric and Process Optimization for Electrical Discharge Machining of Titanium Alloy Ti6Al4V. CIRP J. Manuf. Sci. Technol..

[46] Chaudhari R., Vora J.J., Patel V., Lacalle L.N.L.D., Parikh D.M. (2020). Effect of WEDM Process Parameters on Surface Morphology of Nitinol Shape Memory Alloy. Materials.

[47] Pereira O., Rodríguez A., Fernández-Abia A.I., Barreiro J., López De Lacalle L.N. (2016). Cryogenic and Minimum Quantity Lubrication for an Eco-Efficiency Turning of AISI 304. J. Clean. Prod..

[48] Pereira O., Celaya A., Urbikaín G., Rodríguez A., Fernández-Valdivielso A., Lacalle L.N.L.D. (2020). CO2 Cryogenic Milling of Inconel 718: Cutting Forces and Tool Wear. J. Mater. Res. Technol..

[49] Pereira O., Rodríguez A., Fernández-Valdivielso A., Barreiro J., Fernández-Abia A.I., López-de-Lacalle L.N. (2015). Cryogenic Hard Turning of ASP23 Steel Using Carbon Dioxide. Procedia Eng..

[50] Sliusarenko O., Escudero G.G., González H., Calleja A., Bartoň M., Ortega N., De Lacalle L.N.L. (2023). Constant Probe Orientation for Fast Contact-Based Inspection of 3D Free-Form Surfaces Using (3+2)-Axis Inspection Machines. Precis. Eng..

[51] Muhammad Ashraf W., Moeen Uddin G., Muhammad Arafat S., Afghan S., Hassan Kamal A., Asim M., Haider Khan M., Waqas Rafique M., Naumann U., Niazi S.G. (2020). Optimization of a 660 MWe Supercritical Power Plant Performance—A Case of Industry 4.0 in the Data-Driven Operational Management Part 1. Thermal Efficiency. Energies.

[52] Uddin G.M., Ziemer K.S., Zeid A., Kamarthi S. (2015). Monte Carlo Study of the Molecular Beam Epitaxy Process for Manufacturing Magnesium Oxide Nano-Scale Films. IIE Trans..

[53] Khan A.A. (2008). Electrode Wear and Material Removal Rate during EDM of Aluminum and Mild Steel Using Copper and Brass Electrodes. Int. J. Adv. Manuf. Technol..

[54] Khan M.A.R., Rahman M.M., Kadirgama K. (2015). An Experimental Investigation on Surface Finish in Die-Sinking EDM of Ti-5Al-2.5Sn. Int. J. Adv. Manuf. Technol..

[55] Das S., Paul S., Doloi B. (2020). Feasibility Investigation of Neem Oil as a Dielectric for Electrical Discharge Machining. Int. J. Adv. Manuf. Technol..

[56] Ashraf W.M., Uddin G.M., Arafat S.M., Krzywanski J., Xiaonan W. (2021). Strategic-level performance enhancement of a 660 MWe supercritical power plant and emissions reduction by AI approach. Energy Convers. Manag..

[57] Ashraf W.M., Uddin G.M., Tariq R., Ahmed A., Farhan M., Nazeer M.A., Hassan R.U., Naeem A., Jamil H., Krzywanski J. (2023). Artificial Intelligence Modeling-Based Optimization of an Industrial-Scale Steam Turbine for Moving toward Net-Zero in the Energy Sector. ACS Omega.

[58] Krzywanski J., Blaszczuk A., Czakiert T., Rajczyk R., Nowak W. Artificial intelligence treatment of NOX emissions from CFBC in air and oxy-fuel conditions. Proceedings of the CFB-11: Proceedings of the 11th International Conference on Fluidized Bed Technology.

[59] Ashraf W.M., Uddin G.M., Farooq M., Riaz F., Ahmad H.A., Kamal A.H., Anwar S., El-Sherbeeny A.M., Khan M.H., Hafeez N. (2021). Construction of operational data-driven power curve of a generator by industry 4.0 data analytics. Energies.

[60] Suvarna M., Jahirul M.I., Aaron-Yeap W.H., Augustine C.V., Umesh A., Rasul M.G., Günay M.E., Yildirim R., Janaun J. (2022). Predicting Biodiesel Properties and Its Optimal Fatty Acid Profile via Explainable Machine Learning. Renew. Energy.

[61] Mondal N., Nishant, Ghosh S., Mandal M.C., Pati S., Banik S. (2023). ANN and RSM Based Predictive Model Development and EDM Process Parameters Optimization on AISI 304 Stainless Steel. Mater. Today Proc..

